# Ozone-induced fetal growth restriction in rats is associated with sexually dimorphic placental and fetal metabolic adaptation

**DOI:** 10.1016/j.molmet.2020.101094

**Published:** 2020-10-05

**Authors:** Colette N. Miller, Janice A. Dye, Andres R. Henriquez, Erica J. Stewart, Katelyn S. Lavrich, Gleta K. Carswell, Hongzu Ren, Danielle L. Freeborn, Samantha J. Snow, Mette C. Schladweiler, Judy H. Richards, Prasada R.S. Kodavanti, Anna Fisher, Brian N. Chorley, Urmila P. Kodavanti

**Affiliations:** 1 Public Health and Integrated Toxicology Division, Center for Public Health and Environmental Assessment, Office of Research and Development, US Environmental Protection Agency, 109 T.W. Alexander Dr., Mail Code: B105-02, Research Triangle Park, NC, 27711, USA; 2 Oak Ridge Institute for Science and Education Research Participation Program, US Environmental Protection Agency, 109 T.W. Alexander Dr., Mail Code: B105-02, Research Triangle Park, NC, 27711, USA; 3 Division of the National Toxicology Program, National Institute of Environmental Health Sciences, 530 Davis Dr., Keystone Building, Durham, NC, 27713, USA; 4 Biomolecular and Computational Toxicology Division, Center for Computational Toxicology and Exposure, Office of Research and Development, US Environmental Protection Agency, 109 T.W. Alexander Dr., Mail Code: B105-02, Research Triangle Park, NC, 27711, USA

**Keywords:** Growth restriction, Placenta, Sex differences, Ozone, Developmental origins of health and disease

## Abstract

**Objective:**

The importance of the placenta in mediating the pre- and post-natal consequences of fetal growth restriction has been increasingly recognized. However, the influence of placental sexual dimorphism on driving these outcomes has received little attention. The purpose of this study was to characterize how sex contributes to the relationship between placental metabolism and fetal programming utilizing a novel rodent model of growth restriction.

**Methods:**

Fetal growth restriction was induced by maternal inhalation of 0.8 ppm ozone (4 h/day) during implantation receptivity (gestation days [GDs] 5 and 6) in Long-Evans rats. Control rats were exposed to filtered air. At GD 21, placental and fetal tissues were obtained for metabolic and genomic assessments.

**Results:**

Growth-restricted male placentae exhibited increased mitochondrial biogenesis, increased oxygen consumption, and reduced nutrient storage. Male growth-restricted fetuses also had evidence of reduced adiposity and downregulation of hepatic metabolic signaling. In contrast, placentae from growth-restricted females had elevated markers of autophagy accompanied by an observed protection against hepatic metabolic perturbations. Despite this, growth restriction in females induced a greater number of hypothalamic gene and pathway alterations compared to growth-restricted males.

**Conclusions:**

Increases in mitochondrial metabolism in growth-restricted male placentae likely initiates a sequela of adaptations that promote poor nutrient availability and adiposity. Divergently, the female placenta expresses protective mechanisms that may serve to increase nutrient availability to support fetal metabolic development. Collectively, this work emphasizes the importance of sex in mediating alterations in placental metabolism and fetal programming.

## Highlights

• Peri-implantation exposure to the gaseous air pollutant ozone impairs fetal growth.
• Ozone-induced, growth-restricted male placentae have increased mitochondrial biogenesis and oxidative consumption.
• Female growth-restricted placentae show increased inflammatory and autophagy-like responses.
• Placental metabolic adaptations to growth restriction were associated with sexually dimorphic perturbations in fetal tissues.

## 1 Introduction

Intrauterine growth restriction (IUGR) is a prevalent developmental condition that affects upward of 11% of pregnancies in the US [1] and 24% of births in developing countries [2]. The etiology of IUGR is complex and highly variable. Impaired fetal growth has been associated with poor maternal nutrition, viral infection, and exposures to environmental stressors such as maternal smoking and air pollution [3,4]. Defined as a birth weight below the tenth percentile for gestational age, IUGR is a significant cause of both pre- and perinatal mortality [5]. Moreover, the long-term effects of low birth weight, including increased susceptibility to cardiometabolic disease [6,7], is consistent with the Developmental Origins of Health and Disease (DOHaD) hypothesis.

Sex differences in the risk of IUGR and its consequences have also been reported. While the rates of IUGR are higher overall in female neonates [8], the risk of perinatal mortality is higher in males [9]. This propensity toward greater complications in males extends to childhood and adulthood, particularly in the components of metabolic syndrome [10]. Of these, the most well-studied is hypertension, with restricted growth in utero more closely associated with increased blood pressure in males [11,12]. Similarly, maternal caloric restriction programs male hypersensitivity to post-natal dyslipidemia, insulin resistance, and adiposity [13]. Such observations occur less consistently in females; however, few studies have investigated both sexes concurrently.

Historically, the primary role of the placenta was perceived to serve as a selective passive filter for the exchange of gas, nutrients, and waste between the mother and fetus. However, it is increasingly recognized that the placenta acts more like an orchestrator, readily adapting morphologically and hormonally in response to the maternal and fetal environment [13,14]. Furthermore, sex appears to mediate the flexibility of the placenta to respond to gestational stress. Sexual dimorphism in placental antioxidant and inflammatory defenses [15,16], nutrient reserve capacity [17], and key transcriptional regulatory genes such as O-linked-*N*-acetylglucosamine (O-GlcNAc) transferase (OGT) [18] exists. Hence, placental sex differences have been theorized to be key drivers of dimorphic disease outcomes such as those previously mentioned. For example, reduced placental OGT, which is inherently decreased in males, is linked to impaired neurodevelopment in mice [19].

Placental mitochondrial dysfunction has also been theorized to mediate sexually dimorphic fetal programming due to the mitochondria's importance in resource availability, oxidative stress, and steroidogenesis, as well as their relative abundance in the placenta [20,21]. Numerous studies have reported relationships between pregnancy complications and altered respiratory chain complex activity [[22], [23], [24]]. These mitochondrial adaptations are stratified by sex, with studies demonstrating male placental susceptibility to respiratory complex chain disruption in a guinea pig model of gestational hypoxia [25] and hyperresponsive autophagy in male placentae from obese mothers [26]. While the evidence of sexual dimorphism in the placental adaptation to maternal overnutrition is increasing, little is known about potential sex differences in mitochondrial function during growth restriction. Therefore, the purpose of the current study was to systematically investigate [1] the contribution of sex to placental bioenergetics in a rodent model of growth restriction and [2] the influence of placental sex on subsequent fetal metabolic and hypothalamic gene pathways.

To generate a model of IUGR, pregnant Long-Evans rats were exposed to the gaseous oxidant air pollutant ozone for 4 h on the mornings of gestation days (GDs) 5 and 6, which is the timing of implantation receptivity in the rat [27]. Implantation has been identified as a sensitive window to adverse pregnancy outcomes, as suboptimal trophoblast invasion can impair placentation and uterine and spiral arterial remodeling [28]. Accordingly, acute ozone exposure during implantation receptivity appears to induce fetal growth restriction in part through trophoblast dysfunction [29] and by impeding uterine arterial blood flow during the second half of gestation [16,30,31]. Generally, unhealthy ozone levels have been noted in tropical industrialized areas. Despite the regulation of ground-level ozone in the US, more than 112 million individuals reside in areas that exceed the current National Ambient Air Quality Standard of 0.07 ppm [32]. Considering this, ozone exposure during pregnancy remains a potential risk to placental and fetal development. Herein, we utilized this inducible exposure model to assess the role of placental sex differences in mediating growth restriction-induced alterations of fetal hepatic and hypothalamic development.

## 2 Materials and methods

### 2.1 Generation of the ozone-induced growth-restriction model

As in our previous work, gestation day 1 (plug-positive) Long-Evans rats were received from the local Charles River Laboratory facility (Raleigh, NC, USA) at 12 weeks of age. Dams were singly housed in plexiglass cages in an AAALAC-accredited facility and had ad libitum access to water and a phytoestrogen-free growth and lactation diet (D15092401; Research Diets, Brunswick, NJ, USA). All of the protocols were approved by the Institutional Animal Care and Use Committee of the Center for Public Health and Environmental Assessment at the US Environmental Protection Agency.

The dams were randomly assigned by body weight using an in-house Excel macro to be exposed to either filtered air or 0.8 ppm ozone for 4 h on the mornings (0700-1100) of gestation days 5 and 6 (n = 8–10/group). Ozone was generated using a silent arc discharge generator (OREC) and transferred into Rochester-style Hinners exposure chambers using a mass flow controller. Temperature (∼22.5 °C) and humidity (∼50.0%) were controlled throughout the exposure. The chambers’ ozone concentrations were monitored using an API Model 400 Ozone Analyzer (Teledyne Analytical Instruments, City of Industry, CA, USA).

As previously shown [16,30], this model produces replicable reductions in male and female fetal growth without alterations in total maternal food intake and body weight gain. Nonetheless, peri-implantation ozone exposure at both 0.4 and 0.8 ppm impairs uterine arterial flow in mid-to-late pregnancy in rats. Based on prior investigations, a 4-to-5 fold difference in ^18^O-labeled oxygen incorporation in lavaged bronchoalveolar cells is found between sedentary rats and humans performing intermittent exercise over a 2 h and 0.4 ppm ozone exposure [33]. Hence, the 0.8 ppm concentration used in rats corresponds to 0.16–0.20 ppm in humans.

### 2.2 Necropsy and tissue collection

Fetal and placental endpoints were collected for assessment on GD 21. The dams and fetuses were euthanized using a lethal intraperitoneal dose of pentobarbital (>400 mg/kg). The gravid uterus was dissected and a pooled amnion (amniotic fluid) sample from each dam (n = 8) was collected using a syringe across the entire intact uterus, as accessible volume per fetal sac is minimal in late-stage pregnancy. Amniotic fluid was then centrifuged for 10 min at 2,465×*g* and 4 °C and stored at −80 °C until use. Following the collection of the amnion, the fetuses and placentae were dissected from the uterus, blotted on clean gauze, and weighed. Fetal sex was determined by visual inspection of the anogenital distance and the crown-to-rump lengths of the fetuses were recorded. Fetal weight, length, and placental weight were averaged across the entirety of the litter by sex. For a subset of the fetuses in each litter (the first and second fetuses on the ovarian side of each horn), the liver and hypothalamus were dissected, quick-frozen in liquid nitrogen, and stored at −80 °C until use. To expedite storage, the fetal organs were not weighed before freezing. Finally, unless otherwise specified, in the same subset of fetuses, the decidual layer was removed and the remaining placental tissue was quick-frozen in liquid nitrogen and stored at −80 °C.

### 2.3 Fetal body composition assessment

Lean body mass and fat mass of the non-dissected fetuses were assessed using magnetic resonance as previously described [16,30]. The relative adiposity was calculated by normalizing the fat mass (grams) to lean body mass (grams). For the current analysis, the relative adiposity was then set to the air-exposed, sex-specific control group from each respective study.

### 2.4 Mitochondrial bioenergetics assessment

Mitochondrial respiration was determined utilizing a modified Seahorse coupling assay. Fresh mitochondria were isolated from identified male and female placentae in either the first or second positions on the ovarian side of each horn from n = 4 dams from each exposure group. The selection of the dams and fetuses was performed at random. Following visual sex identification, the decidual layer was removed and discarded, and the remaining placental tissue was gently homogenized using a Dounce homogenizer in ice-cold mitochondrial isolation buffer (MIB) containing 70 mM of sucrose, 210 mM of mannitol, 5 mM of HEPES, and 1 mM of EGTA. Suspended placental tissue was centrifuged at 150×*g* for 15 min at 4 °C. Following aspiration of any lipid layer, the supernatant was collected and centrifuged for 10 min at 10,000×*g* and 4 °C. The supernatant was then removed, and the remaining pellet was washed in ice-cold MIB and again centrifuged. Following two washes, the final pellet was resuspended in 100 uL of MIB + 0.2% (w/v) fatty acid-free BSA and the total protein concentration was assessed using a Pierce BCA Protein Assay Kit (Thermo Fisher Scientific, Waltham, MA, USA). All of the chemicals were purchased from Sigma–Aldrich (St. Louis, MO, USA) unless otherwise specified.

Isolated mitochondria were diluted to 0.08 μg of protein/μL in pre-warmed (37 °C) assay buffer containing 70 mM of sucrose, 220 mM of mannitol, 10 mM of KH2PO4, 3 mM of MgCl2, 2 mM of HEPES, 1 mM of EGTA, 0.6% (w/v) of fatty acid-free BSA, 2 μM of rotenone, and 10 mM of succinate. The oxygen consumption rates of mitochondrial isolates (2 μg) were assessed using a Seahorse XFe96 Analyzer (Agilent Technologies, Santa Clara, CA, USA) during state 3 respiration (ADP, 4 mM), state 4 respiration (Oligomycin, 3.16 μM), and uncoupled state 3 respiration (FCCP, 4 μM). Respiration was arrested using Antimycin A (4 μM). The mix-wait-measure times were 0.5, 0, and 2 min, respectively. Technical replicates of each mitochondrial isolate (n = 5) were included. Excess mitochondrial isolates not used for the Seahorse assay were frozen at −80 °C to assess complex I and IV enzyme activity (Abcam, Cambridge, MA, USA) using a SpectraMax M5 spectrophotometer (Molecular Devices, San Jose, CA, USA).

### 2.5 Mitochondrial density assessments

DNA and RNA from placentae obtained from the first and second uterine horn positions in our previous study [30] were isolated using Quick-DNA and Direct-Zol RNA Purification Kits (Zymo Research, Irvine, CA, USA). Isolated DNA and RNA samples were quantified using a Qubit fluorometer (Thermo Fisher Scientific). To assess the relative density of placental mitochondria, qPCR was performed on isolated DNA for a representative mitochondrial gene (cytochrome c oxidase subunit 1, *mCox1*; f-TGAGCAGGAATAGTAGGGACAG, and r-GGGCTGTGACGATGACATTATAG) and nuclear gene (β-actin, *nActb*; f-GATCGTGAGGAACACTCAGAAG, and r-CACCCTAGGCGGAAAGTTAAG). Mitochondrial biogenesis was assayed using the expression of peroxisome proliferator-activated receptor-γ coactivator 1-α (*Pparγc1α*; Table S1) following reverse transcription with qScript SuperMix (Quanta Biosciences, Gaithersburg, MD, USA). All of the qPCR assays were performed using primers from Integrated DNA Technologies (Coralville, IA, USA) and SYBR Green Master Mix (Thermo Fisher Scientific) on an Applied Biosystems 7900HT Sequence Detection Platform (Thermo Fisher Scientific). The relative quantification was determined from a set of n = 9–10 placentae/sex/group following ΔCt or ΔΔCt calculations from DNA and RNA, respectively. Samples for assay were selected based on the availability of both male and female littermates in the stored subset of tissue across the dams.

For representation, mitochondrial staining was performed on a randomly selected whole male and female placenta from the uterus. Whole placentae were fixed using OCT (Tissue-Tek, Torrance, CA, USA) while freezing over dry ice and stored at −80 °C until use. Placental tissues were sectioned at −16 °C within the sagittal plane at 10 μM thick using a Leica CM1850 microtome (Nussloch, Germany). The sections were incubated for 30 min with 200 mM of MitoTracker Green FM (Thermo Fisher Scientific), which can stain non-live tissue as it binds to cardiolipin and is independent of the membrane potential. Slides were washed with PBS and cover slipped with ProLong Gold Antifade Mountant with DAPI (Thermo Fisher Scientific). The sections were imaged at 20x using identical image settings with a Nikon Ti Widefield Fluorescence Workstation (Melville, NY, USA) equipped with an Andor Zyla sCMOS camera (Oxford Instruments, Abingdon, UK).

### 2.6 Placental and hepatic gene expression

Total RNA from previously frozen placenta (n = 9–10/sex/group) and fetal liver tissue (n = 8/sex/group) obtained from the first or second uterine horn positions were isolated, quantified, and reverse transcribed as described in the previous section. The expression of genes related to autophagy and metabolic signaling (Table S1) was assessed using 15 ng of cDNA by qRT-PCR as previously described. The relative quantification was determined by the ΔΔCt method following the assessment and selection of an appropriately stable endogenous control. *Ppia* and *Rpl13a* were selected for normalization of the placenta and liver, respectively, and were stable in both male and female tissues. When possible, the same fetuses were used for both the placental and fetal tissue endpoints.

### 2.7 Amnion and tissue analysis of metabolic status

Placental and hepatic samples (n = 7–8/sex/group) from fetuses in the first or second uterine horn positions were prepared by homogenizing a section of the frozen whole tissue in ice-cold RIPA buffer containing Pierce protease inhibitor tablets (Thermo Fisher Scientific). Homogenates were centrifuged for 10 min at 10,000×*g* at 4 °C. The supernatant, including the lipid layer, was carefully collected and stored at −80 °C until assessment on a Konelab Arena 30 Clinical Analyzer (Thermo Labsystems, Espoo, Finland). Metabolic endpoints were assayed in either the stored tissue homogenates or amnion fluid (n = 9–10/group) and included free fatty acids (Cell Biolabs, Inc., San Diego, CA, USA), microalbumin (Sekisui Diagnostics, Charlottetown, Prince Edward Island, Canada), protein (Coomassie Plus Protein Assay Kit), blood urea nitrogen (Thermo Fisher Diagnostics, Middletown, VA, USA), and total cholesterol, glucose, and triglycerides (TECO Diagnostics, Anaheim, CA, USA). Glutathione peroxidase was assessed in the amnion fluid as previously described [34]. Because of limited sample recovery and prioritization of metabolic endpoints, the hormones were assessed in a subset of amniotic fluid samples (n = 7–8/group) using a Rat Leptin Insulin Kit from Meso Scale Discovery and read on a MESO QuickPlex SQ 120 system (Meso Scale Diagnostics, Rockville, MD, USA). Placental metabolic endpoints were normalized to the protein concentration and fetal hepatic endpoints were normalized to the input section weight, as an effect of growth restriction was observed on the hepatic protein values.

### 2.8 Placental protein isolation and western blotting

Protein was isolated using ice-cold RIPA buffer containing Pierce Phosphatase and Protease Inhibitor tablets (Thermo Fisher Scientific) from frozen placental tissue (∼75 mg; n = 5/sex/group) from fetuses in the first or second uterine horn positions. Briefly, the tissue was homogenized and centrifuged at 10,000×*g* for 20 min at 4 °C. The supernatant was collected and the total protein concentration was determined using the DC Protein Assay kit (Bio-Rad Laboratories, Inc., Hercules, CA, USA). The samples were maintained at −80 °C until use.

For each sample, 30 μg aliquots of protein were fractionated using SDS-PAGE (4–20% precast polyacrylamide) and transferred to a 12-well nitrocellulose membrane (except for LC3A/B, which was transferred to a PVDF membrane) according to the manufacturer's protocols (Bio-Rad Laboratories, Inc.). Each sex was run on independent membranes. After incubation with 5% BSA in TBST (10 mM of Tris, pH 8.0, 150 mM of NaCl, and 0.5% Tween 20) for 2 h, the membrane was washed once with TBST and incubated overnight with rabbit anti-rat antibodies purchased from Cell Signaling Technology (Danvers, MA, USA): β-actin rabbit mAb (#8457; 1:200), AMPK rabbit mAb (#5832; 1:200), phospho-AMPKα rabbit mAb (#2535; 1:200), Beclin 1 rabbit mAb (#3495; 1:200), LC3A/B antibody (#4108; 1:200), ULK1 rabbit mAb (#8054; 1:800), and phospho-ULK1 antibody (#6888; 1:800). Membranes were washed with TBST and incubated in 5% BSA with a goat anti-rabbit horseradish peroxidase-conjugated secondary antibody (#111-035-003; 1:20000; Jackson ImmunoResearch, West Grove, PA, USA) for 2 h. Following a final wash, blots were detected using SuperSignal West Pico PLUS Chemiluminescent Substrate (Thermo Fisher Scientific). The data were normalized to β-actin during the analysis.

### 2.9 mRNA-seq

Total RNA was isolated using TRIzol with the Direct-Zol RNA Purification Kit (Zymo Research) from whole hypothalami obtained from fetuses in the first or second uterine horn positions. Isolated RNAs were quantified by a Qubit fluorometer (Thermo Fisher), and the quality was checked using a Bioanalyzer (Beckman Coulter, Inc., Santa Clara, CA, USA). All of the samples had a RIN at or above 9. Hypothalamic RNA samples (n = 6/sex/group, 1 μg each) were processed on an Apollo324 system (Takara Bio USA, Inc., Mountain View, CA, USA) using PrepX mRNA selection and mRNA-seq library prep for Illumina. The products from the library preparation were amplified by PCR with the sequencing index primers. The PCR products were purified on the Apollo324 and assessed for quality using the Bioanalyzer. The library concentrations were verified by Qubit. Pooled libraries were then sequenced using a NextSeq 500 sequencer in high-output mode for 75 cycles and single read at a final concentration of 2.0 pM. The average read depth was 20.2 ± 0.5 million reads per sample. Sequencing data were deposited at the National Center for Biotechnology Information Gene Expression Omnibus under accession #GSE156859.

RNA-seq data were first demultiplexed, and adapters and low-quality bases were removed using Partek Flow NGS analysis software (Build 6.0.17.0403, St. Louis, MO, USA). Trimmed reads were aligned to the rat genome (rn6) using STAR 2.5.3a. Raw counts were normalized by counts per million (CPM), adding 0.0001 to all of the normalized counts. Normalized read counts ≤1.0 were removed and log 2 transformed prior to analysis. Differentially expressed genes (DEGs) were determined through gene-specific analysis (GSA) using a multi-model approach. *P* value ≤ 0.05 and log2 (fold change) ≤ −1.3 or ≥ 1.3 cutoffs were applied to identify DEGs. Hierarchical clustering was then generated using average linkages from Euclidean distances in Partek Flow software. The list of DEGs was then used to perform canonical pathway enrichment in the Ingenuity Pathway Analysis (IPA) Knowledgebase (Qiagen, Hilden, Germany). Significantly altered pathways were determined by -log (*P* value) > 1.3.

### 2.10 Verification of sequencing data

Molecules that were associated with the top altered canonical pathways in the male and female growth-restricted fetuses were selected for qRT-PCR verification. In brief, the first and second uterine horn fetal hypothalamic RNA isolates were reverse transcribed (n = 8/sex/group, including the set used for mRNA-seq). Primers associated with serotonin and dopamine signaling and ribosomal protein subunits (Table S1) were designed and assayed by qRT-PCR as previously described. Stable endogenous controls for each sex were identified through the mRNA-seq data and confirmed by a melt curve analysis. *Ppia* and *Ddc* were selected for the females and males, respectively. The ΔΔCt method was used to perform relative quantification and analysis within each sex.

### 2.11 Statistical analysis

Differences between the control (air-exposed) and growth-restricted (ozone-exposed) fetuses within each sex were analyzed by two-sided t-tests for phenotypic and metabolic endpoints and gene- or protein-specific assessment. Wilcoxon's signed-rank test was used for non-parametric data. The data were analyzed and graphed using GraphPad Prism (v6.07; GraphPad, San Diego, CA, USA) and can be found at https://catalog.data.gov. Statistical significance was set at *P* < 0.05.

## 3 Results

### 3.1 Peri-implantation exposure to ozone induces IUGR

As in our previous reports [16,30,31], 0.8 ppm ozone inhalation for 4 h on the mornings of GDs 5 and 6 induced acute reductions in maternal food intake and body weight gain (Table S2). However, peri-implantation ozone exposure had no effect on total gestational food intake, extrauterine body weight gain, or major litter characteristics such as the litter size and sex distribution (Table S2).

**Figure 1:**
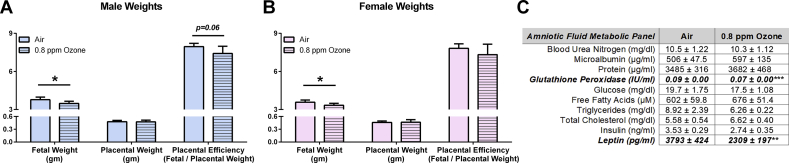
Maternal inhalation of ozone (0.8 ppm) during implantation receptivity (4 h/day, gestation days 5–6) induced a fetal growth-restriction model in Long-Evans rats. Fetal weight was reduced at gestation day 21 in (A) male and (B) female fetuses without alterations in the placental weight of either sex (n = 8 litters per exposure group). (C) Amniotic fluid was obtained from the uterus and pooled for metabolic assessment (n = 9–10 litters per group) and hormones (n = 7–8 litters group). Data are shown as mean ± SEM. Significance levels are derived from two-sided t-testing within each sex. ∗*P* < 0.05, ∗∗*P* < 0.01, and ∗∗∗*P* < 0.001.

Fetal weight was reduced on GD 21 in both sexes (Figure 1A–B). However, the crown-rump length was unaffected in either males (air: 3.87 ± 0.04 cm vs ozone: 3.80 ± 0.04 cm) or females (air: 3.76 ± 0.03 cm vs ozone: 3.70 ± 0.06 cm). Furthermore, we did not observe exposure-induced alterations in relative fetal organ weights (Table S3) or placental weight in either sex (Figure 1A–B). Placental efficiency, which is the ratio of the fetal weight to placental weight and is often calculated in clinical studies as an indicator of maladaptive nutrient transport [35], was also not different between the groups.

Alterations in the metabolic or hormonal profiles of amniotic fluid may be predictive of adverse pregnancy outcomes [36]. Hence, we also sought to characterize changes in the amniotic contents of the ozone-exposed dams. While most endpoints assessed were unaltered by growth restriction, glutathione peroxidase activity was reduced by 22% compared to the control litters (Figure 1C). Furthermore, GD 21 amniotic fluid leptin was reduced by 39%, which may indicate impaired placental efficiency, fetal growth, and adipose tissue development [37]. However, the inability to differentiate this response by sex limits the ability to fully interpret this finding as others have reported both higher amniotic levels and a stronger relationship of leptin to female fetal weight [38,39].

### 3.2 Ozone-induced IUGR disrupts bioenergetics in the male placenta

**Figure 2:**
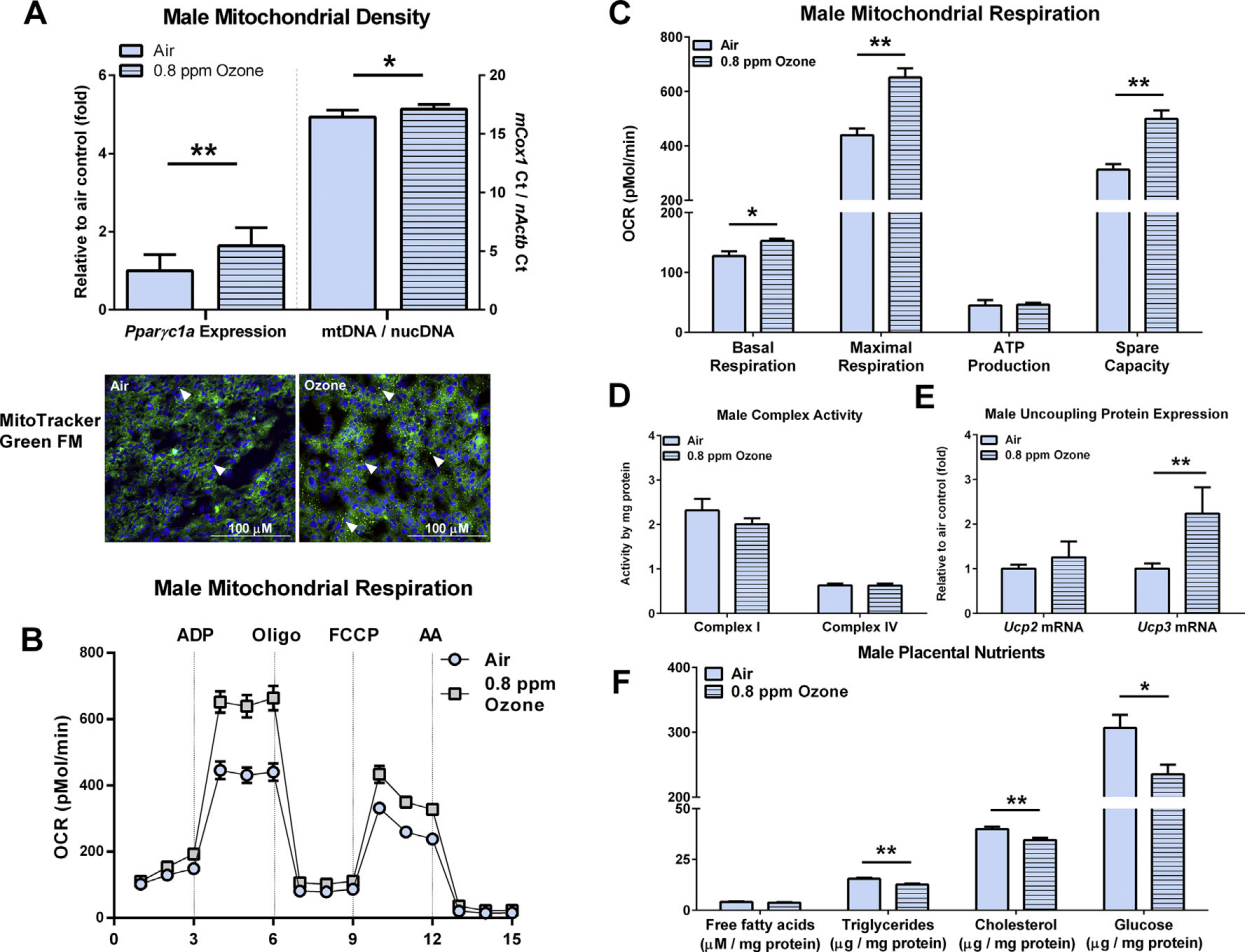
Mitochondrial metabolism was altered in the placentae from the growth-restricted males at gestation day 21. (A) The placentae from the growth-restricted fetuses had increased markers of mitochondrial biogenesis (*Ppargc1α* expression) and abundance as assessed by the expression ratio of mitochondrial DNA (*mCox1*) and nuclear DNA (*nActb*; n = 9–10 litters per group). Mitochondria were stained in a representative OCT-embedded section from each group using MitoTracker Green FM with DAPI nuclear counterstained in blue and imaged at 20x. Arrows point to high-density mitochondrial staining. (B) The oxygen consumption rate of freshly isolated placental mitochondria was assessed by a Seahorse coupling assay (n = 4 litters per group). (C) Oxygen consumption was greater in the placental mitochondria from the growth-restricted males compared to the air-exposed controls. This occurred without changes in (D) complex I and IV activity (n = 8 litters per group), but an (E) increase in *Ucp3* expression (n = 10 litters per group). (F) Placental levels of triglycerides, cholesterol, and glucose (normalized to mg protein) were reduced in the growth-restricted fetuses (n = 8 litters per group). Data are shown as mean ± SEM. Significance levels were derived from two-sided t-testing. ∗*P* < 0.05 and ∗∗*P* < 0.01.

IUGR-induced alterations in placental mitochondrial density have been reported [22,40]; as such, placental mitochondria have been theorized to contribute to both adverse pregnancy outcomes and altered fetal programming. Herein, the relative mitochondrial abundance in the placenta was assessed via the *Pparγc1α* expression and the ratio of mitochondrial DNA (mtDNA) to nuclear DNA (nucDNA). *Pparγc1α* was increased in both the male (Figure 2A) and female (Figure S1A) placentae from the ozone-exposed dams compared to their respective air-exposed controls. The ratio of placental mtDNA/nucDNA was also elevated in the growth-restricted males (Figure 2A) from the ozone-exposed dams compared to the male controls. However, the mtDNA/nucDNA increase within the placentae from the growth-restricted females failed to reach significance compared to the female controls (Figure S1A). Taken together, the data suggest that ozone-induced growth restriction may increase placental mitochondrial density, with greater alterations seen in males.

We next sought to determine if the changes in mitochondrial density were associated with bioenergetic dysfunction in freshly isolated mitochondria from the fetal side of the placenta (labyrinthine region) using the Seahorse coupling assay. Following stimulation of the electron transport chain with ADP or by uncoupling with FCCP (carbonyl cyanide-4-phenylhydrazone), male placentae from the IUGR fetuses had increased oxygen consumption relative to the male placentae from the air-exposed controls (Figure 2B–C). Accordingly, mitochondria from the male IUGR placentae had increases in the basal respiratory state, maximal (ADP-stimulated) respiration, and spare capacity. This upregulation in mitochondrial respiration occurred without increases in either ATP production or complex I and IV activity (Figure 2D). This suggests that elevated oxygen consumption in the male IUGR placenta was not fully attributable to increased cellular energy demands, but likely induced by an increase in proteins involved in respiratory chain uncoupling. Hence, we assessed the expression of the two ubiquitously expressed uncoupling proteins that are linked to mitochondrial redox status (*Ucp2* and *Ucp3*) in addition to the well-studied thermogenic *Ucp1* isoform [41]. While placental *Ucp1* was undetectable and *Ucp2* was unchanged by ozone (Figure 2E), the male growth-restricted placentae had increased *Ucp3* compared to the air-exposed control group. Mitochondrial bioenergetics of the female IUGR placentae remained comparable to that of their corresponding air-exposed controls (Figure S1B–E).

### 3.3 IUGR male fetuses have impaired metabolic status

Due to the elevation in mitochondrial respiration observed in the male IUGR placentae, we next hypothesized that this state of metabolic perturbation would be associated with reduced energy substrate concentrations in the placental labyrinthine. Accordingly, the male IUGR placentae had reductions in triglycerides, cholesterol, and glucose compared to the placentae from the air-exposed controls (Figure 2F). In contrast, no such changes were observed in the female IUGR placentae (Figure S1F).

**Figure 3:**
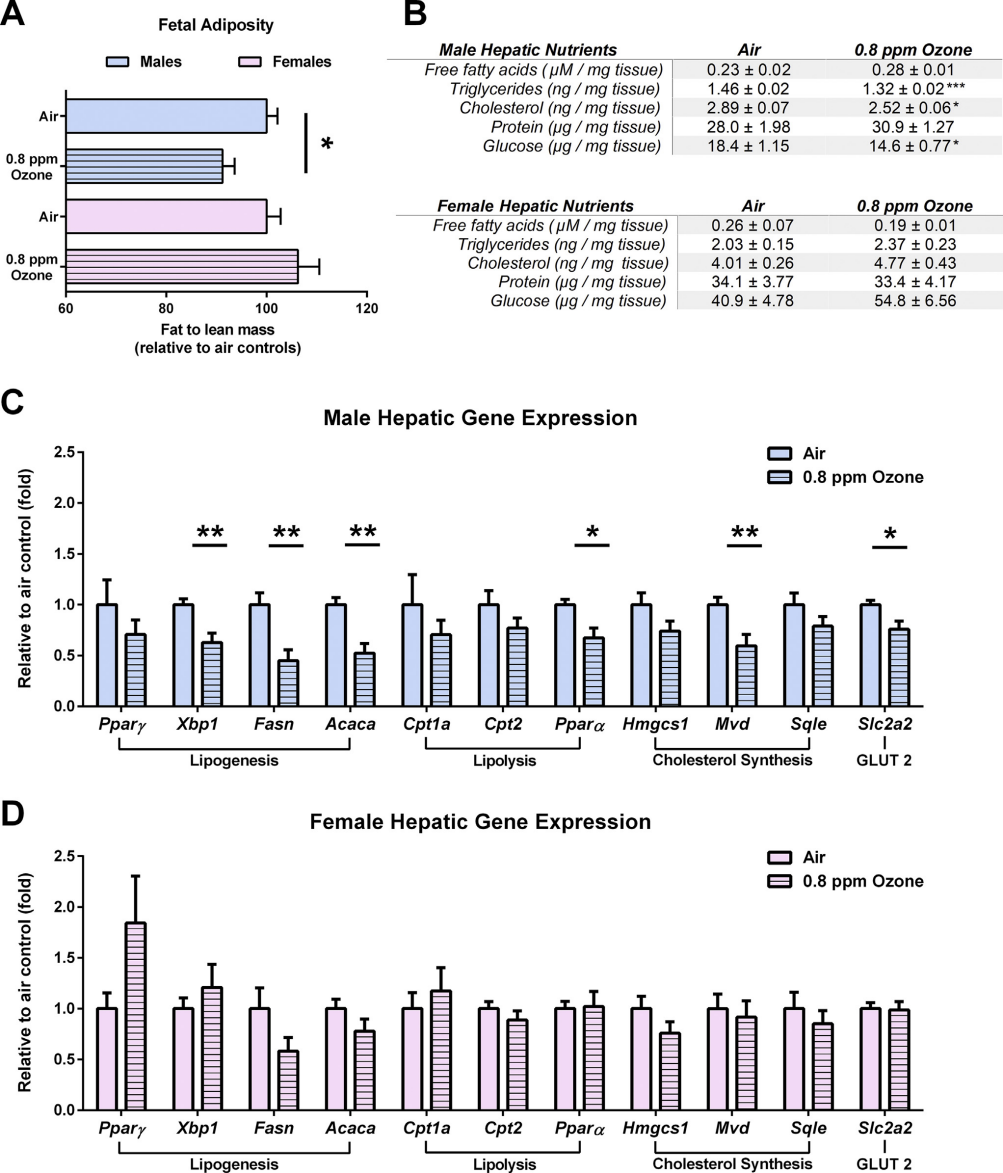
Adiposity and metabolic status in the growth-restricted fetuses at gestation day 21 were sex dependent. (A) Relative whole-body adiposity determined by fat mass (mg)/lean mass (mg) values obtained by magnetic resonance was reduced only the growth-restricted male fetuses compared to the male controls (see Miller et al., 2017 and 2019). (B) Triglycerides, cholesterol, and glucose (normalized to mg of tissue weight) were reduced in the livers from the growth-restricted males compared to the male controls (n = 7–8 litters per group). (C) Hepatic expression of genes related to lipogenesis, lipolysis, cholesterol synthesis, and glucose transport were reduced in the male growth-restricted fetuses compared to the male controls; (D) this was not observed in the female growth-restricted fetuses compared to the female controls (n = 8 litters per group). Data are shown as mean ± SEM. Significance levels were derived from two-sided t-testing within each sex. ∗*P* < 0.05, ∗∗*P* < 0.01, and ∗∗∗*P* < 0.001.

Based on these findings, we next postulated that because the male placentae were utilizing more substrate than necessary, they would in turn provide proportionately less substrate to the fetus, thus limiting male fetal development of lipid stores. To answer this, we investigated differences in the fat-to-lean mass ratio, a measurement of relative adiposity, in GD 21 fetuses of the IUGR and control groups. Relative to lean body mass, the males with IUGR had reduced adiposity compared to the air-exposed controls (Figure 3A). Consequently, the male IUGR fetuses not only weighed less, but were significantly leaner than the controls.

To confirm blunted body fat accrual in the male IUGR fetuses, we next assessed the levels of energy substrates in the fetal liver. Similar to what was observed in the male IUGR placenta, the liver also had reduced levels of triglycerides, cholesterol, and glucose relative to the air-exposed male livers (Figure 3B). Analysis of the gene patterns in the liver further supported an overall blunted metabolic status. Specifically, the male IUGR fetal livers had decreased expression of several lipogenic genes (*Xbp1*, *Fasn*, and *Acaca1*), lipolytic and cholesterol biosynthetic genes (*Pparα* and *Mvd1*), and hepatic glucose transporter *Slc2a2* (Figure 3C). Despite such indices of impaired lipid accrual and hepatic lipogenesis, the relative tissue weight of the liver remained unaltered in the male IUGR fetuses compared to male air-exposed controls (Table S3).

### 3.4 Placental autophagy in IUGR females is associated with improved fetal metabolic status

Females from the ozone-exposed dams did not have a reduced fat-to-lean mass ratio relative to the female control fetuses (Figure 3A). Similar to what was observed in the placentae, the levels of nutrients in the fetal liver appeared to be higher in the females relative to the males (Figure 3B). However, unlike what was found in the IUGR males, neither fetal hepatic energy substrates (Figure 3B) nor the expression of key genes related to hepatic energy metabolism were altered in the female livers from the ozone-exposed dams relative to the female controls (Figure 3D). This suggests that the relative fetal nutrient supply was maintained in the IUGR females despite being physically but proportionately smaller. As previously mentioned, we did not observe changes in mitochondrial bioenergetics in the female placentae attributable to IUGR (Fig. S1). Hence, we next sought to determine if the observed maintenance of fetal adiposity may be related to the sparing of energy within the placenta.

**Figure 4:**
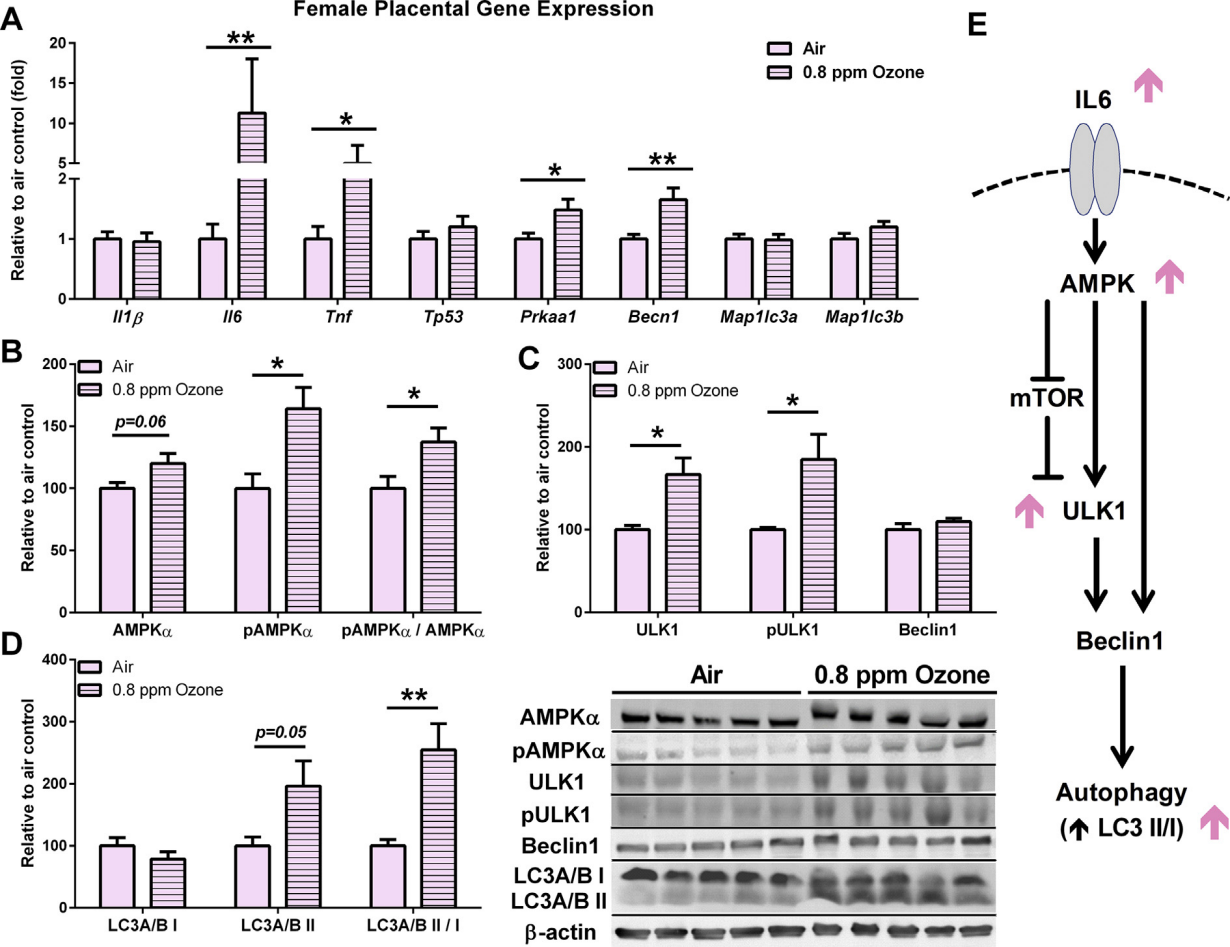
Placentae from the growth-restricted females displayed increased signs of autophagy. (A) The expression of inflammatory cytokines (*Tnfα* and *Il6*) increased in the placentae from the growth-restricted females (n = 9–10 litters per group). This was accompanied by increases in (B) AMPKα and (C) ULK1 phosphorylation and (D) an increased LC3A/B II/I ratio as observed in the accompanying western blotting (n = 5 litters per group). (E) Representative signaling pathway demonstrating how IL6 may regulate autophagy through AMPKα activation. Pink arrows indicate altered protein levels in the female fetuses. Data are shown as mean ± SEM. Significance levels were derived from two-sided t-testing. ∗*P* < 0.05 and ∗∗*P* < 0.01

One such mechanism that the placenta may undertake to reduce its own cellular energy requirements is autophagy, which is known to be activated during times of stress (for example, hypoxia, changes in resource availability, and inflammatory injury) [42]. As a critical regulator of autophagy, AMPKα controls numerous cellular pathways that promote autophagosome development, reduce inflammatory signaling, and suppress protein synthesis. Herein, the IUGR female placentae had increased AMPKα transcript (*Prkaa1*) and phosphorylated AMPKα levels, as well as increases in the ratio of phosphorylated AMPKα to total compared to the air controls (Figure 4A–B). Importantly, a similar upregulation was not observed in the male IUGR placentae (Figure S2A–B).

To confirm whether autophagic signaling downstream of AMPKα activation occurred, we next assessed differential expression markers of autophagy in the IUGR placentae of both sexes. While few differences were observed between the male placentae (Fig. S2), numerous indicators of autophagic signaling increased in the placentae of the IUGR females. Both total ULK1 and phosphorylated ULK1 were upregulated in the female IUGR placentae compared to the female controls (Figure 4C). Furthermore, LC3II, a key phosphatidylethanolamine-conjugated protein involved in autophagosome expansion and accumulation, increased in the IUGR females. This change resulted in an increase in the LC3II/LC3I ratio. An over 5-fold increase in the expression of both *Tnfα* and *Il6* was measured in the female IUGR placenta (Figure 4A), both of which are also possible inducers of autophagy [43]. While autophagic flux was not assessed, taken together, these data indicate that ozone-induced growth restriction corresponds with signs of autophagy activation in the female, but not male, placenta (Figure 4D).

### 3.5 IUGR is associated with altered hypothalamic gene expression in both sexes

**Figure 5:**
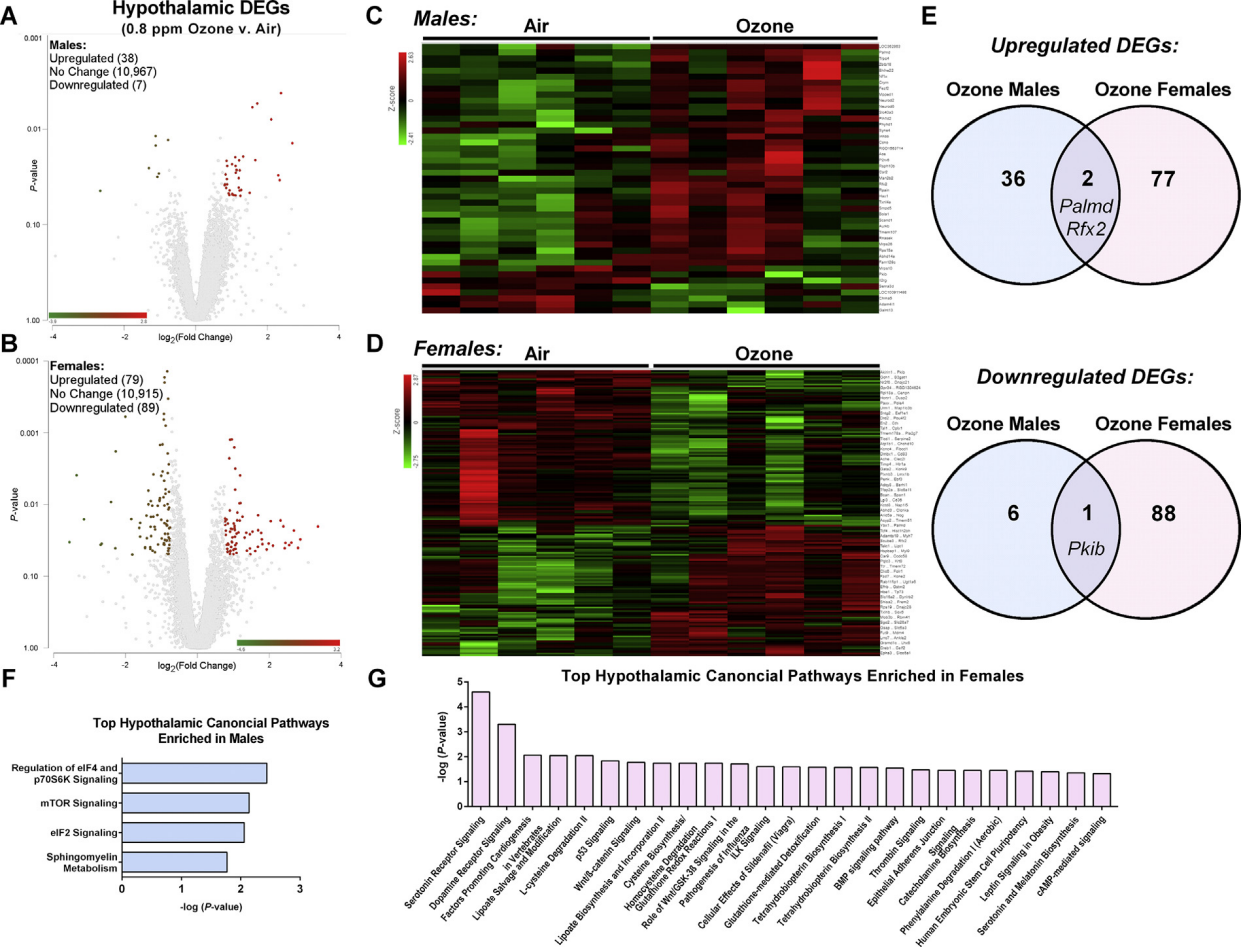
Ozone-induced growth restriction results in sexually dimorphic alterations in hypothalamic gene expression at gestation day 21. mRNA-seq was performed on whole hypothalamic fetal tissue (n = 6 litters per group). (A–B) Volcano plots and heatmaps (C–D) of differentially expressed genes (DEGs) in the male and female samples were defined using cutoffs at p ≤ 0.05 and log_2_ (fold change) ≤ −1.3 or ≥ 1.3 from controls. (E) The growth-restricted females had both a larger number and unique set of upregulated and downregulated DEGs compared to the growth-restricted males. Ingenuity pathway analysis was then used to identify significantly altered pathways (-log (*P* value) > 1.3) in the growth-restricted (F) males and (G) females.

IUGR is associated with an increased risk of obesity in offspring, likely driven in part by reprogramming of the hypothalamus due to suboptimal nutrition and hypoxia during development [44]. As such, we performed mRNA-seq on whole hypothalamic tissue to identify altered expression of genes in our IUGR model and major pathways that could be associated with fetal undernutrition near term. Of nearly 11,000 genes measured by mRNA-seq, relatively few differentially expressed genes (DEGs) attributable to growth restriction were found in either sex (Figure 5 A-D and Tables S4–S5). Nonetheless, growth restriction in the females resulted in 79 upregulated and 89 downregulated genes, whereas IUGR in the males resulted in 38 upregulated and 7 downregulated DEGs compared to their respective controls. Only three of the identified DEGs shared directional similarity between sexes: *Palmd*, *Rfx2*, and *Pkib* (Figure 5E).

We next performed ingenuity pathway analysis (IPA) from the list of DEGs to identify canonical pathways associated with IUGR in hypothalamic tissue. Although the males expressed far fewer pathway disruptions compared to the females (4 vs 25), the top significant pathways changed with IUGR in the males included the regulation of eIF4 and p70S6K signaling, mTOR signaling, and eIF2 signaling (Figure 5F). The genes most associated with these pathways were mainly ribosomal proteins, including *Rps10*, *Rps15a*, and *Rps26* (Table S6). In the females, the top two pathways that changed with IUGR were serotonin receptor signaling and dopamine receptor signaling (Figure 5G), with corresponding genes such as *Htr1a*, *Ddc*, and *Drd2* being associated with these pathways (Table S7). Using this information, a small subset of genes was selected to be validated using qRT-PCR. Overall, this analysis confirmed our mRNA-seq findings (Fig. S3).

## 4 Discussion

Within the context of fetal growth restriction, few studies have characterized the potential relationship between placental sex and corresponding fetal metabolic signaling. Nonetheless, we hypothesized that placental sex is a significant contributing factor to the near-term outcomes observed in our model of ozone-induced IUGR, changes that are consistent with differential cardiometabolic disease risk. Prenatal exposure to air pollution is an emerging risk factor for the development of IUGR [4]. In our studies, we have consistently reported an enhanced male severity to growth restriction induced by peri-implantation inhalation to the gaseous air pollutant ozone [16,30,31]. Because of the exposure timing and chemistry of ozone, suppressed fetal growth is unlikely to be due to direct embryonic exposure to ozonation products (for example, lipid peroxides). However, the mechanisms that link acute exposure to ozone early in pregnancy to impaired fetal growth remain unknown. We previously demonstrated both impaired invasiveness in trophoblasts cultured in serum from ozone-exposed dams [29] and alterations in uterine arterial resistance in mid-to-late gestation following peri-implantation ozone exposure [30], both of which implicate the likely involvement of placental hypoxia and nutrient restriction in driving fetal growth impairment. As such, we sought to determine whether placentation adapts differentially by sex in response to acute exposure to ozone in early pregnancy. Furthermore, we investigated if such alterations may be related to disruptions in fetal hepatic and hypothalamic development.

We focused on alterations in placental mitochondrial bioenergetics due to the important roles that mitochondria play in nutrient and oxygen utilization. IUGR has been associated with both increases [22,24,40] and decreases [45,46] in placental mitochondrial content. In our growth-restriction model, mitochondrial density markers were elevated in the placental tissue from both sexes. Taken together, this suggests that a relationship between placental mitochondrial function and IUGR could be driven by multiple factors including fetal sex, environmental stress, and placental cell type.

Conversely, in the face of growth restriction, the female placentae did not exhibit altered mitochondrial respiration, whereas the placentae from the growth-restricted males were more metabolically active and capable of producing ATP under stimulation (for example, spare capacity). By extension, the reduced levels of placental triglycerides, cholesterol, and glucose in the male growth-restricted placentae were consistent with the observed increase in mitochondrial respiration. Although we do not know the mechanism for this difference, numerous regulators of mitochondrial metabolism have shown differential responses between the sexes. In isolated female syncytiotrophoblasts, TNFα stimulation decreased mitochondrial respiration [47], which serves as a protective adaptation to reduce oxidative stress [48]. Male syncytiotrophoblasts, however, were not able to mount a similar response, consistent with the concept that the placenta's metabolic flexibility is different between the sexes [47].

In contrast to the high metabolic capacity and demand of the male placenta in the current study, autophagy markers were upregulated in the female placenta. Autophagy is a cellular defense mechanism that may serve to protect basal cellular function in the face of nutrient deprivation and/or during inflammatory stress [42]. Thus, predictably, elevated levels of autophagy have been observed in placentae from pregnancies complicated with preeclampsia and in rodent models of acute maternal food deprivation [42,49]. While the role of autophagy remains an area of active investigation [50], placental-specific deletion of ATG7 has been shown to be associated with an elevated risk of cardiometabolic disease in male offspring [26]. Our findings likewise support some of Muralimanoharan et al.’s conclusions [26], in that the female placenta during growth restriction may also engage in autophagy as a compensatory mechanism to recycle nutrients and secure substrate availability to support fetal growth.

The growth-restricted male fetuses in our study had reduced adiposity and decreased hepatic levels of triglycerides and cholesterol. Accordingly, the expression of genes related to lipid metabolism in the liver was also suppressed. Importantly, this reduction in adiposity indices in the male fetuses from the ozone-exposed dams was independent of changes in maternal food intake. The growth-restricted females, however, did not demonstrate signs of an impaired metabolic state relative to the air-exposed female fetuses. In fact, the female fetal liver appeared to have higher levels of substrates such as cholesterol and triglycerides compared to the male liver. Similar observations have been reported in swine, with female fetuses demonstrating increased levels of hepatic lipogenesis, allantoic fluid cholesterol, and placental essential free fatty acid transfer [[51], [52], [53]]. Further, these studies provide evidence that females maintain the capacity to support metabolic development in swine models of undernutrition, an effect not observed in male fetuses. Taken together, we suggest that the observed sexual dimorphism found herein was attributable to sex differences in placental energy utilization and transfer. However, it is unclear why the male placentae from our growth-restriction model had an upregulated metabolic state in the first place.

One theory to explain this paradoxical phenomenon is that male placenta may have increased nutrient transporters compared to females and that these transporters serve to increase substrate availability to support enhanced fetal growth in males. While sexual dimorphism in human placental nutrient transporters has not yet been comprehensively described across all types, increasing evidence suggests that placental sex drives how nutrient transporters adapt to adverse environments. Elevated placental fatty acid transporters in males have been reported in rodent models of both maternal high-fat and high-salt consumption [54] and thromboxane A2-induced gestational hypertension [55]. Such increases, however, may be problematic to the placenta as increased lipids can result in lipotoxicity, a phenomenon that has been described in placentae from obese mothers [56].

To circumvent lipotoxicity, the cell may upregulate mitochondrial respiratory chain uncoupling to expend excess lipids, thus limiting superoxide formation [57]. In the current study, the placental mitochondria from the IUGR males failed to produce higher levels of ATP despite an increase in oxygen consumption. This finding was indicative of elevated uncoupling, thus, the observed increase in *Ucp3* transcript in the IUGR males was not surprising. Moreover, *Ucp3* is responsive to the formation of lipid peroxides and may act as an adaptive fatty acid mitochondrial exporter [57]. Hence, the elevation in the *Ucp3* transcript levels in the IUGR males herein supports the plausibility of a placental lipotoxic state and the activation of mechanisms to protect against excess lipids. As the male sex has shown greater UCP3 responsiveness in lipotoxic conditions [58], the noted *Ucp3* increase may help explain not only the reductions in placental substrates observed in IUGR males but may also be involved in the blunting of substrate levels in males.

Male fetuses grow at a faster pace than females in utero [17]. Thus, we hypothesize that the concentrations of varying transporters in the placenta not only differ by sex but are also likely to adapt to subsequent stressors differentially according to the sex of the fetus. While our model is not one of maternal undernutrition, based on our previous work demonstrating impaired uterine blood flow in mid-to-late gestation [16,30,31], we suspect that nutrient availability to the placenta may be blunted. We propose that the placentae from males may adapt to increase nutrient transport in the face of fetal growth restriction and/or impaired blood supply, rendering them more susceptible to lipotoxicity unless compensatory efforts to expend excess lipids are not upregulated (for example, fatty acid oxidation). Importantly, we did not herein assess anaerobic metabolism, which may also serve to counteract placental lipotoxicity. It is plausible that the protection observed in the female placenta may have been driven by a preference for glycolysis. Autophagy has been positively associated with anaerobic metabolism in cancer cells [59]. Hence, it is likewise plausible that the autophagic activation observed in IUGR females may predicate a glycolytic shift. While sex differences in placental glycolytic capacity were not observed in embryonic day 18 mice [60], there are few studies that investigate placental glycolysis in mediating the sex-specific adaptation in adverse maternal environments.

Despite a sexually dimorphic alteration in the metabolic status of both the placenta and fetal liver in this fetal growth-restriction model, the observed hypothalamic perturbation due to maternal peri-implantation ozone exposure was minimal. Nonetheless, the total number of both DEGs and pathways altered in the fetuses from the ozone-exposed dams were greater in the females than in the males (DEG: 168 vs 45; pathways: 24 vs 4). While it is difficult to ascertain why hypothalamic gene expression appears to be more greatly altered in IUGR females, sex differences in the relative rate of neurodevelopment may contributed to this observation [61]. Furthermore, non-nutritive placental factors/signals disrupted in growth-restricted females (for example, placental-derived cytokines) may also serve to impair optimal neurodevelopment independently of metabolic fetal status. While placental cytokine transfer is highly time- and species-dependent, placental immunomodulatory proteins have been theorized to mediate post-natal neurobehavioral disease [62]. Future work is necessary to better understand the role of placental dysfunction in neurodevelopment and the programming of metabolism. However, considering the evidence shown herein, it may be hypothesized that the relevant placental mediators will vary depending on the fetal tissue system in question.

While further studies are needed to firmly establish a link between changes in bioenergetics in the male placenta and disturbances in fetal metabolic status, the co-occurrence of these phenomena suggest that they are related. Importantly, placental mitochondria are structurally and functionally diverse within the organ's regions and these mitochondrial subpopulations may also respond differently depending on gestational age [20,63]. Additional work is needed to elucidate how sex and specific placental cell populations interact to support fetal development. Nevertheless, the relatively consistent findings in each tissue investigated and within each sex provide evidence that placental energy metabolism likely differs between growth-restricted males and females. To conclude, our work highlights the importance of studying sex differences in developmental outcomes and provides initial evidence that sex differences in placental bioenergetics may contribute to both IUGR pathogenesis and fetal metabolic programming.

## Disclaimer

This project was supported in part by an appointment to the Research Participation Program at the Center for Public Health and Environmental Assessment at the US EPA administered by the Oak Ridge Institute for Science and Education through an interagency agreement between the US Department of Energy and US EPA. This article may be the work product of an employee or group of employees of the National Institute of Environmental Health Sciences (NIEHS) at the National Institutes of Health. The research described in this article was reviewed and approved for publication by NIEHS and the Center for Public Health and Environmental Assessment at the US EPA. Approval does not signify that the contents reflect the views or policies of the US EPA, the Department of Health and Human Services, nor the US government. The mention of trade names or commercial products does not constitute endorsement or recommendation for use.
