# Supplementary material for: Ozone-induced fetal growth restriction in rats is associated with sexually dimorphic placental and fetal metabolic adaptation
Source: Mol Metab. 2020 Oct 5;42:101094. doi: 10.1016/j.molmet.2020.101094 (PMC7588867; doi:10.1016/j.molmet.2020.101094)
Supplement: Multimedia component 1 [file mmc1.docx]

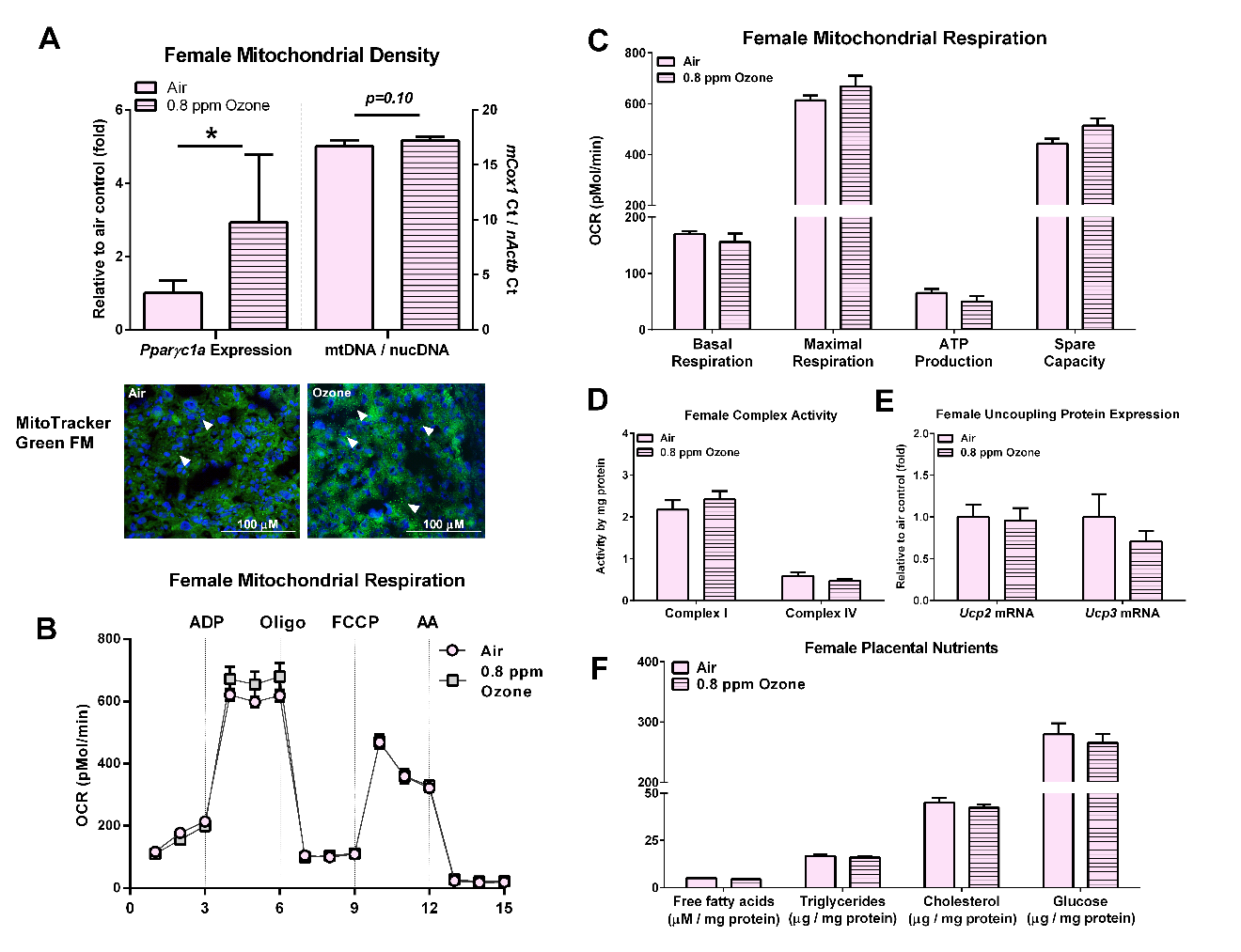


Fig. S1. Mitochondrial metabolism is not altered in female growth restricted placentas at gestation day 21. (A) The placentas from growth restricted offspring have increased markers of mitochondrial biogenesis (*Ppargc1α* expression) and abundance as assessed by the expression ratio of mitochondrial DNA (*mCox1*) and nuclear DNA (*nActb*; n = 10 litters per group). Mitochondria were stained in a representative OCT embedded section from each group using MitoTracker Green FM with DAPI nuclear counter stain in blue and imaged at 20x. Arrows point to high density mitochondrial staining. (B) The oxygen consumption rate of freshly isolated placental mitochondria was assessed by a Seahorse Coupling Assay (n = 4 litters per group). (C) Oxygen consumption, (D) Complex I and IV activity (n = 8 litters per group), or (E) expression of uncoupling proteins (n = 10 litters per group) were not altered in growth restricted female placentas. (F) Placental levels of triglycerides, cholesterol, and glucose (normalized to mg protein) were also not altered in growth restricted offspring (n = 7 – 8 litters per group). Data are shown as mean ± SEM. Significance levels are derived from two-sided t-testing. **P* < 0.05.

Fig. S2. Placentas from growth restricted males do not display signs of autophagy. (A) The expression of inflammatory cytokines and autophagy markers in male placentas (n = 9 – 10 litters per group). No alterations in (B) AMPKα, (C) ULK1, or (D) LC3A/B post-translational modification were observed, as shown in the accompanying western blot (n = 5 litters per group). (E) Representative signaling pathway demonstrating how IL6 may regulate autophagy through AMPK activation. The blue arrow indicates altered protein levels in male offspring. Data are shown as mean ± SEM. Significance levels are derived from two-sided t-testing. **P* < 0.05.


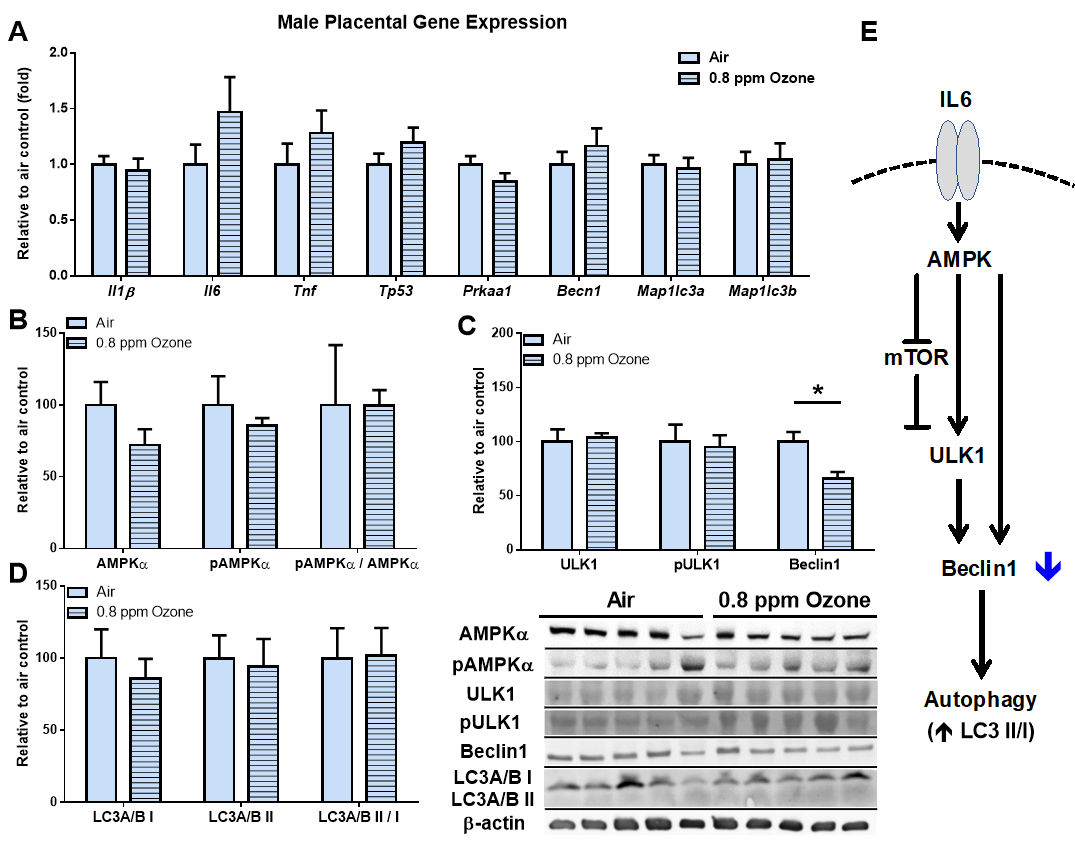


Figure S3. Confirmation of RNA-seq findings. A subset of hypothalamic genes identified from the top canonical pathways altered in the RNA-seq data were assayed by qRT-PCR (n = 8 litters per group) in (A) males and (B) females. Data are shown as mean ± SEM. Significance levels are derived from two-sided t-testing within each sex. **P* < 0.05, ****P* < 0.001.


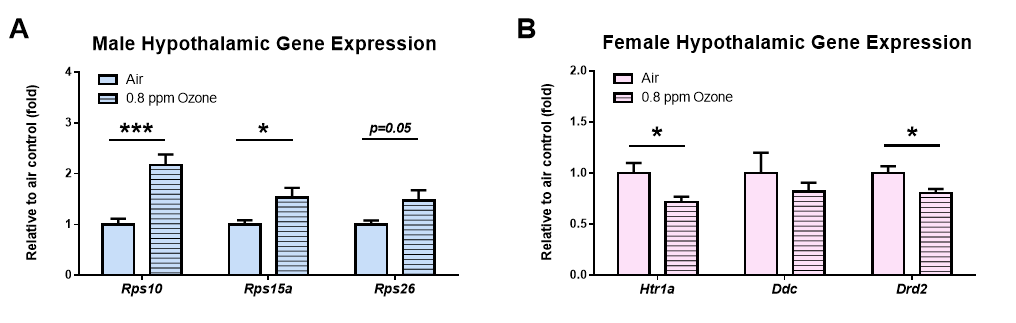


Table S1. List of qRT-PCR primers

| **Gene ID** | **Forward** | **Reverse** |
| --- | --- | --- |
| *Acaca* | ACTGTGAGGTGGATCAGAGA | CAGGCTCCAGATGTCGATAAAT |
| *Becn1* | CTCAGGAGAGGAGCCATTTATT | CCCGATCAGAGTGAAGCTATT |
| *Cpt1a* | GCTGCATGGAAGATGCTTTG | CCAGGAGTGCTCTGCATTTA |
| *Cpt2* | CCTACCTGGTCAACGCATATC | GTCTAGCCTTGGTGTCAGTAAA |
| *Ddc* | CATAAAGCCGTAGGTGGTAGAA | GCCACACAGAGGACAAGTTA |
| *Drd2* | GCTCACCCTCCTCATCTTTATC | CAGCAAGGCTGACTATCAAGTA |
| *Fasn* | CAACCGGCTCTCTTTCTTCTT | GCCTGATAGGCATTCTGTAGTG |
| *Hmgcs1* | AAGGCTAGTGCTGAGCTATTC | GGAACCGTATACAGAGGATGTG |
| *Htr1a* | GTCACCTGCGACCTGTTTAT | TAGTCTATAGGGTCGGTGATAGC |
| *Il1β* | CTTCCTAAAGATGGCTGCACTA | CTGACTTGGCAGAGGACAAA |
| *Il6* | GAAGTTAGAGTCACAGAAGGAGTG | GTTTGCCGAGTAGACCTCATAG |
| *Map1lc3a* | GCACAGCATGGTGAGTGTAT | GAAGGTTTCTTGGGAGGCATAG |
| *Map1lc3b* | GTCCGAGAAGACCTTCAAACA | TCTCACCCTTGTATCGCTCTA |
| *Mvd* | GCTGATCCTGCCCATCAA | CCTCTGTGAAGTCCTTGCTAAT |
| *Ppia* | CAAAGTTCCAAAGACAGCAGAAA | CTGTGAAAGGAGGAACCCTTATAG |
| *Pparα* | CGGAAACTGCAGACCTCAAA | GCTAGTCTTTCCTGCGAGTATG |
| *Pparγ1* | TGACCACTCCCATTCCTTTG | GTGAACGGGATGTCTTCATAGT |
| *Pparγc1α* | GCCGGAGCAATCTGAGTTAT | GATCACCAAACAGCCGTAGA |
| *Prkaa1* | TCCTTTCTGGTGTGGACTATTG | CGGCTATCTTTGCATTCATGTG |
| *Rpl13a* | CTGTGAGGGCATCAACATTTC | GTTGGTGTTCATCCGCTTTC |
| *Rps10* | AAGAACCGGATTGCCATCTAC | GGTTGGGAACGTTCTTGTCT |
| *Rps15a* | TCAGGCCGTGTTCCAAAG | CCCAGCTCTGTGATCATCAAT |
| *Rps26* | TGCGTGCCCAAGGATAAG | CGAAGACGCTTGCTTCAGATA |
| *Slc2a2* | TCATAGTCACACCAGCACATAC | AGACAGAGACCAGAGCATAGT |
| *Sqle* | TTCGCTGCCTTCTCAGATATTC | GGACTCCAGCTGTTCTTTCTT |
| *Tnf* | ACCTTATCTACTCCCAGGTTCT | GGCTGACTTTCTCCTGGTATG |
| *Tp53* | GGAGGATTCACAGTCGGATATG | CTGTGGTGGGCAGAATATCA |
| *Ucp1* | CTAGCAGACATCATCACCTTCC | GATGGTCCCTAAGACACCTTTATAC |
| *Ucp2* | GAACGGGACACCTTTAGAGAAG | CAATCGTCAAGACGAGACAGAG |
| *Ucp3* | TGTGGTGAAGGTCCGATTTC | GGTTCTGTAGGCATCCATAGTC |
| *Xbp1* | GACTGCGCGAGATAGAAAGAA | CTAGCTGGAGTTTCTGGTTCTC |

Primers were designed using sequence information for Rattus norvegicus derived from the National Center of Biotechnology Information and purchased through Integrated DNA Technologies, Inc.

Table S2. Effect of peri-implantation ozone exposure on maternal weight, food intake, and litter characteristics.

| Characteristic | Air | 0.8 ppm Ozone |
| --- | --- | --- |
| Peri-implantation food intake (g) | 56.7 ± 3.35 | 43.6 ± 1.52** |
| Peri-implantation body weight gain (g) | 5.91 ± 1.69 | -4.15 ± 1.67*** |
| Total food intake (g) | 517 ± 21.4 | 513 ± 17.9 |
| Extrauterine weight gain (g) | 162 ± 8.38 | 153.2 ± 7.99 |
| Implantation sites per litter | 13.0 ± 0.80 | 13.6 ± 0.78 |
| Resorptions per litter | 0.50 ± 0.27 | 1.25 ± 0.45 |
| Total fetuses | 12.5 ± 0.68 | 12.8 ± 1.09 |
| Percent males per litter | 41.4 ± 3.66% | 51.5 ± 3.54% |

Food intake and body weight gain were measured daily starting on gestation day 3. Dams were exposed for 4 hours on gestation days 5 and 6 during implantation receptivity. Peri-implantation food intake and body weight gain derived from the difference in food and body weight on gestation day 5 and 7. Extrauterine weight gain is the difference in body weight between gestation day 21 and 5. Litter characteristics were obtained at time of necropsy on gestation day 21. Data are shown as mean ± SEM (n = 8 litters per group). Significance levels are derived from two-sided t-testing. **P < 0.01, ***P < 0.001.

Table S3. Relative fetal organ weights at gestation day 21.

| Tissue | Air | 0.8 ppm Ozone |
| --- | --- | --- |
| Male liver  (mg/mg body weight) | 0.045 ± 0.002 | 0.043 ± 0.002 |
| Male hypothalamus  (mg/mg body weight) | 0.010 ± 0.001 | 0.010 ± 0.001 |
| Female liver  (mg/mg body weight) | 0.042 ± 0.002 | 0.042 ± 0.002 |
| Female hypothalamus  (mg/mg body weight) | 0.010 ± 0.001 | 0.012 ± 0.001 |

Organs were dissected from fetuses at the 1^st^ and 2^nd^ uterine horn position nearest the ovary at time of necropsy and immediately frozen. Weights were obtained when tissues were thawed for assay. Data are shown as mean ± SEM (n = 6 and 8 litters per group for liver and hypothalamus, respectively).

Table S4. Differentially expressed genes in the male hypothalamus.

| **Gene ID** | **0.8 ppm Ozone Average** | **Air Average** | **Fold Change from Air** | ***P*-value** |
| --- | --- | --- | --- | --- |
| ***Neurod2*** | 3.14 | 1.23 | 2.55 | 0.01 |
| ***Fezf2*** | 2.06 | 0.90 | 2.28 | 0.00 |
| ***Neurod6*** | 13.67 | 6.06 | 2.25 | 0.03 |
| ***Bhlhe22*** | 12.40 | 5.57 | 2.22 | 0.03 |
| ***Crym*** | 7.32 | 3.52 | 2.08 | 0.01 |
| ***Rpain*** | 4.09 | 2.26 | 1.81 | 0.01 |
| ***Zbtb18*** | 23.08 | 12.97 | 1.78 | 0.02 |
| ***Nf1x*** | 24.91 | 14.39 | 1.73 | 0.01 |
| ***Rps10*** | 18.21 | 10.80 | 1.69 | 0.05 |
| ***Fam129c*** | 6.41 | 4.05 | 1.58 | 0.02 |
| ***Bola1*** | 1.89 | 1.22 | 1.55 | 0.04 |
| ***RGD1563714*** | 1.71 | 1.11 | 1.54 | 0.05 |
| ***Ccno*** | 3.44 | 2.24 | 1.53 | 0.03 |
| ***Scand1*** | 4.15 | 2.71 | 1.53 | 0.04 |
| ***Rnasek*** | 16.66 | 10.95 | 1.52 | 0.02 |
| ***Rps26*** | 17.83 | 11.76 | 1.52 | 0.03 |
| ***Smpd5*** | 1.62 | 1.07 | 1.52 | 0.04 |
| ***Car2*** | 12.57 | 8.45 | 1.49 | 0.03 |
| ***Trpc4*** | 8.31 | 5.60 | 1.48 | 0.03 |
| ***Mpped1*** | 27.89 | 18.92 | 1.47 | 0.03 |
| ***Palmd*** | 7.67 | 5.21 | 1.47 | 0.05 |
| ***Tmem107*** | 10.59 | 7.20 | 1.47 | 0.02 |
| ***Hax1*** | 4.01 | 2.74 | 1.47 | 0.02 |
| ***Abhd14a*** | 3.82 | 2.64 | 1.44 | 0.05 |
| ***Txnl4a*** | 5.34 | 3.75 | 1.42 | 0.05 |
| ***Aurkb*** | 13.70 | 9.62 | 1.42 | 0.05 |
| ***Slc43a3*** | 3.92 | 2.76 | 1.42 | 0.02 |
| ***Rsph10b*** | 8.58 | 6.18 | 1.39 | 0.04 |
| ***Syne4*** | 2.37 | 1.71 | 1.38 | 0.05 |
| ***Ace*** | 13.57 | 9.86 | 1.38 | 0.05 |
| ***LOC362863*** | 1.96 | 1.44 | 1.36 | 0.02 |
| ***Rfx2*** | 2.78 | 2.05 | 1.35 | 0.04 |
| ***Pih1d2*** | 1.57 | 1.16 | 1.35 | 0.03 |
| ***Man2b2*** | 17.09 | 12.67 | 1.35 | 0.02 |
| ***Rps15a*** | 9.25 | 6.89 | 1.34 | 0.04 |
| ***Inhbb*** | 5.55 | 4.16 | 1.33 | 0.03 |
| ***P2rx6*** | 4.92 | 3.71 | 1.33 | 0.04 |
| ***Phyhd1*** | 2.57 | 1.94 | 1.32 | 0.04 |
| ***Galnt13*** | 10.04 | 13.11 | -1.31 | 0.01 |
| ***Adam4l1*** | 2.20 | 3.14 | -1.43 | 0.03 |
| ***Sema3d*** | 4.74 | 6.86 | -1.45 | 0.03 |
| ***Chrna5*** | 2.38 | 3.50 | -1.47 | 0.01 |
| ***Il2rg*** | 0.84 | 1.23 | -1.47 | 0.01 |
| ***Pkib*** | 1.20 | 1.89 | -1.57 | 0.03 |
| ***LOC100911498*** | 1.19 | 3.00 | -2.52 | 0.04 |

Table S5. Differentially expressed genes in the female hypothalamus.

| **Gene ID** | **0.8 ppm Ozone Average** | **Air Average** | **Fold Change from Air** | ***P*-value** |
| --- | --- | --- | --- | --- |
| ***Folr1*** | 4.40 | 1.38 | 3.20 | 0.02 |
| ***Ttr*** | 181.08 | 67.06 | 2.70 | 0.03 |
| ***Kl*** | 7.15 | 2.71 | 2.64 | 0.04 |
| ***Clic6*** | 14.74 | 5.81 | 2.54 | 0.02 |
| ***Tmem72*** | 5.36 | 2.16 | 2.48 | 0.03 |
| ***Rprml*** | 3.10 | 1.28 | 2.42 | 0.04 |
| ***Mfrp*** | 14.90 | 6.37 | 2.34 | 0.03 |
| ***Car12*** | 3.84 | 1.65 | 2.32 | 0.04 |
| ***Hbe1*** | 2.08 | 0.91 | 2.29 | 0.02 |
| ***Ugt1a6*** | 4.93 | 2.21 | 2.23 | 0.02 |
| ***Kcne2*** | 8.82 | 4.26 | 2.07 | 0.04 |
| ***Lhx8*** | 6.78 | 3.34 | 2.03 | 0.02 |
| ***Car9*** | 1.78 | 0.90 | 1.99 | 0.03 |
| ***Krt8*** | 2.11 | 1.08 | 1.95 | 0.04 |
| ***Sostdc1*** | 7.56 | 4.03 | 1.87 | 0.04 |
| ***Serpinb1a*** | 3.76 | 2.02 | 1.86 | 0.03 |
| ***Lhx6*** | 9.88 | 5.34 | 1.85 | 0.02 |
| ***Rab11fip1*** | 5.43 | 3.02 | 1.80 | 0.02 |
| ***Tp73*** | 4.86 | 2.70 | 1.80 | 0.01 |
| ***Slc26a7*** | 1.74 | 0.97 | 1.79 | 0.04 |
| ***Slc5a5*** | 2.63 | 1.51 | 1.75 | 0.04 |
| ***Shisa2*** | 1.50 | 0.88 | 1.70 | 0.02 |
| ***Gstm2*** | 2.08 | 1.25 | 1.67 | 0.05 |
| ***Pqlc3*** | 1.40 | 0.84 | 1.66 | 0.04 |
| ***Tcf4*** | 22.91 | 13.81 | 1.66 | 0.03 |
| ***Rin1*** | 1.47 | 0.91 | 1.61 | 0.04 |
| ***Prtg*** | 1.94 | 1.21 | 1.60 | 0.02 |
| ***Lepr*** | 3.08 | 1.95 | 1.58 | 0.03 |
| ***Efhb*** | 1.87 | 1.21 | 1.55 | 0.01 |
| ***Hspbap1*** | 3.84 | 2.54 | 1.52 | 0.01 |
| ***Frem2*** | 10.52 | 6.96 | 1.51 | 0.00 |
| ***Mob3b*** | 2.44 | 1.62 | 1.50 | 0.02 |
| ***Zfp458*** | 2.84 | 1.89 | 1.50 | 0.01 |
| ***Dynlrb2*** | 3.75 | 2.52 | 1.49 | 0.02 |
| ***Fut9*** | 4.55 | 3.09 | 1.47 | 0.01 |
| ***Gstt2*** | 3.48 | 2.37 | 1.47 | 0.02 |
| ***Sgo2*** | 1.77 | 1.21 | 1.46 | 0.03 |
| ***Myl9*** | 2.00 | 1.37 | 1.46 | 0.05 |
| ***Usp2*** | 1.79 | 1.23 | 1.46 | 0.03 |
| ***Myh7*** | 1.46 | 1.01 | 1.45 | 0.04 |
| ***Sox6*** | 4.74 | 3.28 | 1.44 | 0.00 |
| ***Dnajc28*** | 1.64 | 1.13 | 1.44 | 0.04 |
| ***Mpp7*** | 3.23 | 2.25 | 1.44 | 0.01 |
| ***Fzd7*** | 4.07 | 2.83 | 1.44 | 0.03 |
| ***Creb1*** | 2.70 | 1.88 | 1.44 | 0.02 |
| ***Epha3*** | 14.69 | 10.26 | 1.43 | 0.02 |
| ***Tekt1*** | 5.59 | 3.90 | 1.43 | 0.02 |
| ***Rbm41*** | 7.70 | 5.38 | 1.43 | 0.04 |
| ***Slc16a2*** | 17.48 | 12.24 | 1.43 | 0.00 |
| ***Adamts19*** | 4.03 | 2.84 | 1.42 | 0.02 |
| ***Pou2f1*** | 3.47 | 2.45 | 1.42 | 0.05 |
| ***Palmd*** | 8.82 | 6.23 | 1.42 | 0.04 |
| ***Atp6ap1l*** | 1.82 | 1.30 | 1.40 | 0.03 |
| ***Hist1h2bh*** | 7.28 | 5.21 | 1.40 | 0.02 |
| ***Gsap*** | 2.00 | 1.43 | 1.40 | 0.03 |
| ***Ccnt1*** | 7.18 | 5.15 | 1.39 | 0.00 |
| ***Gramd1c*** | 3.94 | 2.83 | 1.39 | 0.03 |
| ***Sox5*** | 6.23 | 4.50 | 1.39 | 0.04 |
| ***Ccdc58*** | 1.61 | 1.17 | 1.38 | 0.02 |
| ***Rps19*** | 15.86 | 11.53 | 1.38 | 0.04 |
| ***Trim5*** | 4.85 | 3.53 | 1.37 | 0.04 |
| ***Celf2*** | 58.91 | 42.93 | 1.37 | 0.00 |
| ***Txlnb*** | 2.09 | 1.53 | 1.37 | 0.02 |
| ***Lipt1*** | 1.22 | 0.89 | 1.37 | 0.05 |
| ***Slc5a3*** | 8.37 | 6.12 | 1.37 | 0.01 |
| ***Slco5a1*** | 7.58 | 5.57 | 1.36 | 0.00 |
| ***LOC654482*** | 16.71 | 12.39 | 1.35 | 0.04 |
| ***Scube3*** | 5.41 | 4.03 | 1.34 | 0.04 |
| ***Mdm4*** | 19.33 | 14.40 | 1.34 | 0.02 |
| ***Mid1*** | 6.53 | 4.87 | 1.34 | 0.05 |
| ***Rfx2*** | 2.89 | 2.16 | 1.34 | 0.05 |
| ***Lysmd3*** | 3.28 | 2.46 | 1.33 | 0.04 |
| ***Zhx2*** | 5.15 | 3.87 | 1.33 | 0.04 |
| ***Ltbp1*** | 10.03 | 7.57 | 1.33 | 0.02 |
| ***Dclre1c*** | 3.24 | 2.46 | 1.32 | 0.02 |
| ***St18*** | 7.19 | 5.47 | 1.32 | 0.03 |
| ***Csnk1g1*** | 8.18 | 6.25 | 1.31 | 0.02 |
| ***Zfp397*** | 7.36 | 5.63 | 1.31 | 0.02 |
| ***Lrrc7*** | 10.44 | 7.99 | 1.31 | 0.02 |
| ***Paxx*** | 14.55 | 18.93 | -1.30 | 0.02 |
| ***Plekhb1*** | 6.42 | 8.36 | -1.30 | 0.03 |
| ***RGD1304624*** | 4.57 | 5.98 | -1.31 | 0.03 |
| ***Rassf5*** | 8.78 | 11.50 | -1.31 | 0.04 |
| ***Gmnn*** | 5.71 | 7.48 | -1.31 | 0.04 |
| ***Pbx3*** | 52.97 | 69.53 | -1.31 | 0.02 |
| ***Cth*** | 3.45 | 4.52 | -1.31 | 0.01 |
| ***B3gat1*** | 121.99 | 160.26 | -1.31 | 0.01 |
| ***Kctd8*** | 21.14 | 27.85 | -1.32 | 0.00 |
| ***Tmem178a*** | 6.46 | 8.52 | -1.32 | 0.03 |
| ***Chchd10*** | 4.20 | 5.54 | -1.32 | 0.05 |
| ***Serpine2*** | 83.53 | 110.42 | -1.32 | 0.01 |
| ***Rpl18a*** | 106.60 | 140.93 | -1.32 | 0.04 |
| ***Acyp2*** | 3.67 | 4.87 | -1.32 | 0.04 |
| ***Pole4*** | 35.61 | 47.20 | -1.33 | 0.00 |
| ***Oxtr*** | 4.98 | 6.62 | -1.33 | 0.03 |
| ***Acot1*** | 20.05 | 26.68 | -1.33 | 0.00 |
| ***Atp1b1*** | 538.57 | 716.75 | -1.33 | 0.00 |
| ***Hs3st5*** | 13.49 | 17.97 | -1.33 | 0.01 |
| ***Slc6a11*** | 58.62 | 78.33 | -1.34 | 0.02 |
| ***Qdpr*** | 45.02 | 60.19 | -1.34 | 0.00 |
| ***Ddc*** | 13.90 | 18.59 | -1.34 | 0.05 |
| ***Mfsd3*** | 9.01 | 12.05 | -1.34 | 0.02 |
| ***Psmd10*** | 5.77 | 7.74 | -1.34 | 0.03 |
| ***Dnajc21*** | 8.09 | 10.86 | -1.34 | 0.02 |
| ***Cenpn*** | 7.17 | 9.65 | -1.35 | 0.03 |
| ***Spon1*** | 65.07 | 87.90 | -1.35 | 0.02 |
| ***Map1lc3b*** | 109.52 | 148.72 | -1.36 | 0.00 |
| ***Ppp4c*** | 43.30 | 58.84 | -1.36 | 0.01 |
| ***Cd83*** | 11.26 | 15.30 | -1.36 | 0.01 |
| ***Syndig1l*** | 4.03 | 5.51 | -1.37 | 0.03 |
| ***Tlcd1*** | 2.52 | 3.45 | -1.37 | 0.03 |
| ***Nap1l5*** | 229.44 | 314.85 | -1.37 | 0.00 |
| ***Urm1*** | 34.27 | 47.05 | -1.37 | 0.00 |
| ***Gpr34*** | 1.19 | 1.64 | -1.37 | 0.02 |
| ***Kcnc4*** | 36.13 | 49.64 | -1.37 | 0.01 |
| ***Adcy8*** | 15.81 | 21.72 | -1.37 | 0.04 |
| ***Fam212b*** | 21.03 | 28.97 | -1.38 | 0.00 |
| ***Pkib*** | 1.39 | 1.92 | -1.38 | 0.03 |
| ***Nr2f6*** | 20.25 | 27.96 | -1.38 | 0.00 |
| ***Ache*** | 60.63 | 83.81 | -1.38 | 0.00 |
| ***Drd2*** | 4.36 | 6.04 | -1.38 | 0.04 |
| ***Syt17*** | 26.51 | 36.75 | -1.39 | 0.01 |
| ***Gdpd2*** | 39.35 | 55.04 | -1.40 | 0.00 |
| ***Abhd3*** | 16.21 | 22.68 | -1.40 | 0.00 |
| ***Acot4*** | 2.05 | 2.89 | -1.41 | 0.03 |
| ***Sntg2*** | 1.11 | 1.57 | -1.42 | 0.02 |
| ***Clec2l*** | 13.00 | 18.42 | -1.42 | 0.00 |
| ***Lin7b*** | 7.00 | 10.02 | -1.43 | 0.00 |
| ***Eef1e1*** | 62.12 | 89.16 | -1.44 | 0.01 |
| ***Fibcd1*** | 6.81 | 9.80 | -1.44 | 0.01 |
| ***Ybx1*** | 69.19 | 99.66 | -1.44 | 0.01 |
| ***Gch1*** | 1.49 | 2.17 | -1.46 | 0.04 |
| ***Gpr37l1*** | 3.55 | 5.21 | -1.47 | 0.01 |
| ***Phlda3*** | 3.22 | 4.73 | -1.47 | 0.01 |
| ***Lgi3*** | 12.49 | 18.78 | -1.50 | 0.02 |
| ***Myorg*** | 3.04 | 4.58 | -1.50 | 0.05 |
| ***Hcrtr1*** | 1.61 | 2.43 | -1.51 | 0.02 |
| ***Vwa5b2*** | 18.31 | 27.97 | -1.53 | 0.00 |
| ***Tal1*** | 19.88 | 30.39 | -1.53 | 0.01 |
| ***Bcan*** | 45.08 | 69.44 | -1.54 | 0.04 |
| ***Nog*** | 1.86 | 2.88 | -1.55 | 0.01 |
| ***Hsd11b1*** | 1.06 | 1.66 | -1.56 | 0.03 |
| ***Mag*** | 2.50 | 3.92 | -1.56 | 0.01 |
| ***Timp4*** | 4.51 | 7.06 | -1.57 | 0.01 |
| ***Ebf3*** | 89.24 | 140.09 | -1.57 | 0.02 |
| ***Tmem51*** | 1.10 | 1.74 | -1.57 | 0.04 |
| ***Cplx1*** | 42.54 | 67.01 | -1.58 | 0.04 |
| ***Arid5a*** | 1.26 | 2.02 | -1.60 | 0.01 |
| ***Srxn1*** | 1.49 | 2.40 | -1.60 | 0.01 |
| ***Lynx1*** | 11.56 | 18.80 | -1.63 | 0.03 |
| ***Htr1a*** | 0.86 | 1.40 | -1.63 | 0.01 |
| ***Lmx1b*** | 3.17 | 5.24 | -1.65 | 0.04 |
| ***Gata2*** | 2.60 | 4.30 | -1.66 | 0.04 |
| ***Clcnka*** | 3.55 | 6.16 | -1.74 | 0.02 |
| ***Plxnb3*** | 1.49 | 2.59 | -1.74 | 0.03 |
| ***Ebf2*** | 8.26 | 14.46 | -1.75 | 0.02 |
| ***Kcnk9*** | 4.46 | 8.35 | -1.87 | 0.05 |
| ***Cd36*** | 1.33 | 2.52 | -1.89 | 0.02 |
| ***Myh6*** | 1.12 | 2.24 | -2.00 | 0.00 |
| ***Tfap2b*** | 12.56 | 27.60 | -2.20 | 0.00 |
| ***Penk*** | 4.38 | 9.67 | -2.21 | 0.04 |
| ***Barhl1*** | 2.81 | 6.46 | -2.30 | 0.01 |
| ***Pou4f2*** | 2.15 | 5.42 | -2.52 | 0.04 |
| ***En2*** | 4.66 | 11.86 | -2.54 | 0.04 |
| ***Pla2g7*** | 1.27 | 3.78 | -2.98 | 0.02 |
| ***Dmbx1*** | 1.10 | 3.51 | -3.20 | 0.00 |
| ***Mab21l2*** | 10.72 | 36.79 | -3.43 | 0.03 |
| ***Tfap2a*** | 1.19 | 5.51 | -4.64 | 0.02 |

Table S6. IPA results from differential gene expression in male hypothalamus.

| **Ingenuity Canonical Pathways** | **-log(*P*-value)** | **Ratio** | **Z-Score** | **Molecules** |
| --- | --- | --- | --- | --- |
| Regulation of eIF4 and p70S6K Signaling | 2.44 | 0.0205 |  | RPS26, RPS10, RPS15A |
| mTOR Signaling | 2.14 | 0.016 |  | RPS26, RPS10, RPS15A |
| EIF2 Signaling | 2.06 | 0.0149 |  | RPS26, RPS10, RPS15A |
| Sphingomyelin Metabolism | 1.77 | 0.125 |  | SMPD5 |

**Table S7. IPA results from differential gene expression in female hypothalamus.**

| **Ingenuity Canonical Pathways** | **-log(*P*-value)** | **Ratio** | **Z-Score** | **Molecules** |
| --- | --- | --- | --- | --- |
| Serotonin Receptor Signaling | 4.59 | 0.128 |  | GCH1, HTR1A, DDC, ADCY8, QDPR |
| Dopamine Receptor Signaling | 3.3 | 0.0694 |  | GCH1, DRD2, DDC, ADCY8, QDPR |
| Factors Promoting Cardiogenesis in Vertebrates | 2.06 | 0.0449 |  | MYH7, TCF4, FZD7, NOG |
| Lipoate Salvage and Modification | 2.04 | 1 |  | LIPT1 |
| L-cysteine Degradation II | 2.04 | 1 |  | CTH |
| p53 Signaling | 1.83 | 0.0385 | -1 | SERPINE2, TP73, KL, MDM4 |
| Wnt/β-catenin Signaling | 1.77 | 0.0305 | 0 | SOX6, SOX5, TCF4, CSNK1G1, FZD7 |
| Lipoate Biosynthesis and Incorporation II | 1.74 | 0.5 |  | LIPT1 |
| Cysteine Biosynthesis/Homocysteine Degradation | 1.74 | 0.5 |  | CTH |
| Glutathione Redox Reactions I | 1.74 | 0.087 |  | GSTT2/GSTT2B, GSTM1 |
| Role of Wnt/GSK-3β Signaling in the Pathogenesis of Influenza | 1.71 | 0.0476 |  | TCF4, CSNK1G1, FZD7 |
| ILK Signaling | 1.6 | 0.0275 | 1.342 | MYH7, CREB1, MYL9, KL, MYH6 |
| Cellular Effects of Sildenafil (Viagra) | 1.59 | 0.0325 |  | MYH7, MYL9, MYH6, ADCY8 |
| Glutathione-mediated Detoxification | 1.58 | 0.0714 |  | GSTT2/GSTT2B, GSTM1 |
| Tetrahydrobiopterin Biosynthesis I | 1.57 | 0.333 |  | GCH1 |
| Tetrahydrobiopterin Biosynthesis II | 1.57 | 0.333 |  | GCH1 |
| BMP signaling pathway | 1.54 | 0.0411 |  | CREB1, SOSTDC1, NOG |
| Thrombin Signaling | 1.47 | 0.0254 | 1.342 | GATA2, CREB1, MYL9, KL, ADCY8 |
| Epithelial Adherens Junction Signaling | 1.45 | 0.0294 |  | MYH7, TCF4, MYL9, MYH6 |
| Catecholamine Biosynthesis | 1.45 | 0.25 |  | DDC |
| Phenylalanine Degradation I (Aerobic) | 1.45 | 0.25 |  | QDPR |
| Human Embryonic Stem Cell Pluripotency | 1.42 | 0.0288 |  | TCF4, KL, FZD7, NOG |
| Leptin Signaling in Obesity | 1.39 | 0.0357 |  | LEPR, KL, ADCY8 |
| Serotonin and Melatonin Biosynthesis | 1.35 | 0.2 |  | DDC |
| cAMP-mediated signaling | 1.32 | 0.023 | -0.447 | DRD2, HTR1A, CREB1, ADCY8, PKIB |
